# Supplementary material for: Single-shot link discovery for terahertz wireless networks
Source: Nat Commun. 2020 Apr 24;11:2017. doi: 10.1038/s41467-020-15761-4 (PMC7181786; doi:10.1038/s41467-020-15761-4)
Supplement: Supplementary file 1 — Supplementary Information [file 41467_2020_15761_MOESM1_ESM.pdf]

Single-shot link discovery for terahertz wireless networks

Yasaman Ghasempour[1], Rabi Shrestha[2], Aaron Charous[2], Edward Knightly[1], and Daniel M. Mittleman[2*]

*Corresponding author: daniel_mittleman@brown.edu

Supplementary materials

Supplementary Note 1. Discussion of the two models

Our ray optics approach, defined by Eq. 3 of the main text, is derived from simple geometric considerations. With reference to Fig. 1b, we introduce additional notation: we denote the distance between the two LWGs along the *z* axis as Z_0 and along the y axis as Y_0, such that $R^2 = Z_0^2 + Y_0^2$ (where *R* is defined in the figure). We can write two expression for cos ϕ_0: (i) from geometry we have $\cos \phi_0 = Y_0/R$, and (ii) from wave propagation we have $\cos \phi_0 = k_y/k_0$. Here k_0 is the free space wave vector and k_y is its *y* component. Combining these two expressions, we can write

$$Y_0 = \frac{k_y}{k_0} R \tag{1}$$

Similarly, we can describe Z_0 by

$$Z_0 = \frac{k_z}{k_0} R \tag{2}$$

Simple geometric considerations allow us to find:

$$\tan\theta_{\min} = \frac{Z_0}{Y_0 + L} \qquad \tan\theta_{\max} = \frac{Z_0}{Y_0 - L} \tag{3}$$

After substituting Y_0 and Z_0 using Supplementary equations (1) and (2) and some simplifications, we can derive Eq. (3) of the main text.

The above derivation is a simple ray-optics description which relies on knowledge of the (finite) length of the slot apertures in the two leaky-wave devices (both the TX and the RX have the same slot length L). In contrast, the underlying description of Eq. (2) of the main text involves diffraction through an aperture of finite length, and thus relies only on the length of the TX aperture, not the RX aperture. In this sense, the two models are not equivalent in the general case; in order to render them equivalent, one would need to account for the finite aperture of the RX in the diffraction formalism. This could be accomplished, for example, by convolving the diffraction pattern of Eq. (2) of the main text with an aperture of finite size which subtends a finite angular range with respect to the TX waveguide; this would have the effect of broadening the spectrum that would be predicted at any given angle, relative to what is shown in Fig. 2b of the main text. However, in our case, the effects of this broadening would be minimal, since the TX-RX distance (R = 15 cm) is significantly larger than the slot length (L = 3 cm) (so therefore $\alpha L \approx 3.7$). This claim is substantiated by the good agreement between the measured spectra (see Fig. 2a of the main text) and the spectra computed directly from Eq. (2) of the main text (see Fig. 2b of the main text).

Supplementary Note 2. Discussion of client rotation

To understand the changes in the received spectrum that result from a rotation of the client (i.e., a non-zero value of θ_{rot}), we may use a similar ray-based approach to predict the frequency distribution at a specific angle, given the geometry of transmitting and receiving waveguides, and their separation. We denote θ_{min} and θ_{max} as the minimum and maximum angles a light ray could be received by a LWG with slot length L and propagation distance R (see Fig. 1b of the main text). We consider a RX LWG which is parallel to, and at angle ϕ_0 with respect to, the transmit LWG. In such case, the AoD of rays radiating out of the transmit LWG is

the same as their AoA when impinging on the receiver LWG; thus, they couple into the waveguide with minimum coupling loss at all radiated frequencies. Hence, as discussed above, we measure a spectral band whose low-frequency and high-frequency edges are determined by ϕ_0. In particular, the high frequency and low-frequency contours are similar to the two dotted black curves presented in Fig. 2b of the main text.

Figure S1 illustrates the key components of our rotation estimation model. We denote the low-frequency and high-frequency edges of this initial spectral band (when there is zero rotation) as $f_{\min,0}$ and $f_{\max,0}$, respectively. Then, we measure the changes in this spectral region of interest in order to detect clockwise (CW) or counter clockwise (CCW) rotation. Specifically:

- CW rotation: In the case, the effective AoA of impinging rays increases by the amount of rotation (θ_{rot}). Hence, the larger frequency components experience higher coupling loss causing a reduction in the high-frequency edge of the measured spectrum. For a given rotation angle θ_{rot}, of a receiver located in parallel and in the far field of TX LWG, the spectral change is described by

$$\Delta f\left(\theta_{\text{rot}}\right) = f_{\max,\theta_{\text{rot}}} - f_{\max,0} = \left[\left. \frac{\partial f_{\max}\left(\theta\right)}{\partial \theta} \right|_{\phi_0} \right] \theta_{\text{rot}} \tag{4}$$

  where $f_{\max}(\theta)$ is the max-frequency contour shown as a solid black curve in Fig. S1. Note that the $f_{\min}$ of the measured spectrum is expected to remain the same with a CW rotation. The reason is, as shown in Fig. S1, the initial low-frequency edge ($f_{\min,0}$) falls into the spectral band of larger angles (i.e., $\phi_0 + \theta_{rot}$). Also, the receiver is blind to any spectral change beyond the initial spectral region of interest; hence, frequencies beyond the range [$f_{\min,0} - f_{\max,0}$] are not received.

- CCW rotation: Conversely, when rotated counterclockwise, the AoA of the impinging

waves change to $\phi_0 - \theta_{rot}$ causing high coupling loss for lower frequency components in the spectral region of interest. Similarly, we relate the amount of rotation to the changes in the low-frequency edge ($f_{\min}$) of the measured spectrum as follows:

$$\Delta f(\theta_{rot}) = f_{\min,\theta_{rot}} - f_{\min,0} = \left[\frac{\partial f_{\min}(\theta)}{\partial \theta} \bigg|_{\phi_0} \right] \theta_{rot} \qquad (5)$$

As above, the $f_{\min}$ of the measured spectrum after a CCW rotation is expected to remain the same and $f_{\min}(\theta)$ is the low-frequency contourobtained via the ray optics model.

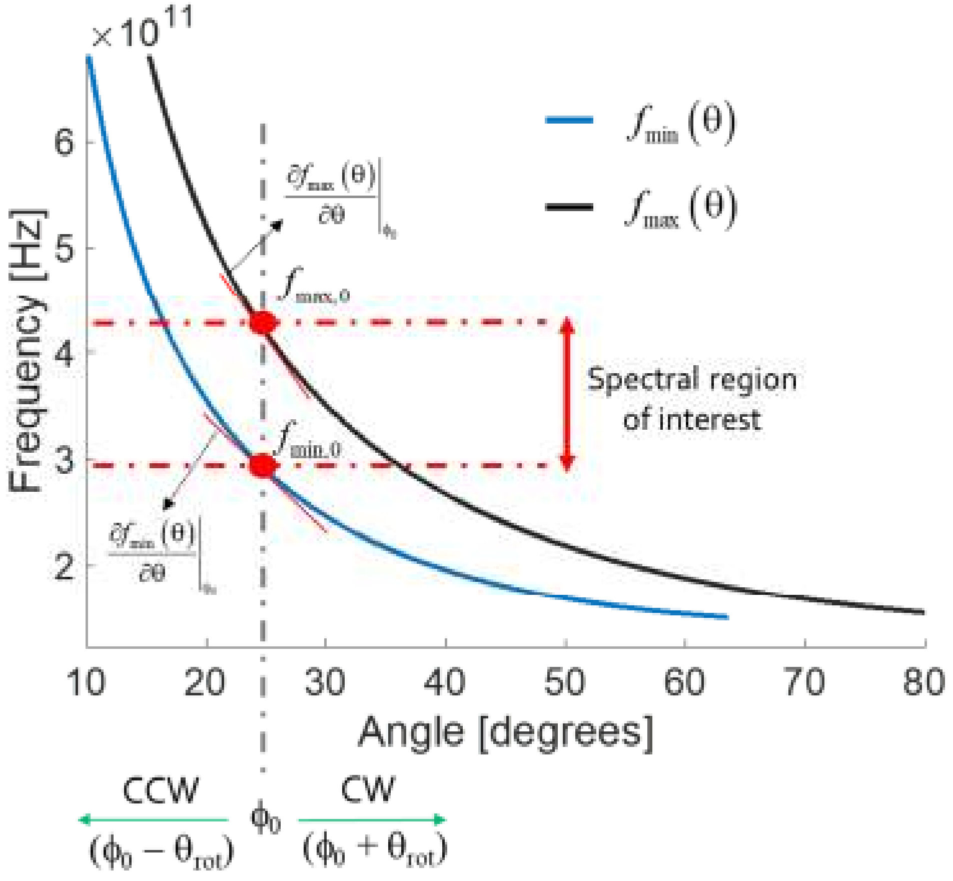

Figure S1 – The key components of our rotation estimation model. The two curves show the high-frequency and low-frequency edges of the spectral band. The quantities $f_{\min,0}$ and $f_{\max,0}$ are the low-frequency and high-frequency edges of this initial spectral band (when there is zero

rotation), respectively. We estimate the amount of rotation (θ_{rot}) by tracking the changes in the spectral width of the received spectrum. A CW rotation causes a decrease in the high-frequency edge of received spectrum while a CCW rotation results in an increase of the low-frequency edge.
